# Neuroborreliosis as a Cause of Acute Ischemic Stroke in a 13-Year-Old Patient

**DOI:** 10.7759/cureus.91374

**Published:** 2025-08-31

**Authors:** Anna Sendor, Lukasz Wentrys, Lidia Stopyra

**Affiliations:** 1 Department of Infectious and Tropical Diseases, Andrzej Frycz Modrzewski Krakow University Medical College, Krakow, POL; 2 Department of Infectious Diseases and Pediatrics, Specialist Zeromski Hospital, Krakow, POL; 3 Department of Infectious and Tropical Diseases, Andrzej Frycz Modrzewski Krakow University Medical College, Kraków, POL

**Keywords:** ceftriaxone therapy, cerebrospinal fluid, children, complication, cytosis, facial paresis, ischemic stroke, lyme disease, mri, neuroborreliosis

## Abstract

Acute ischemic stroke is a rare condition in the pediatric population. This case highlights the importance of considering neuroborreliosis as a potential cause of stroke in children, emphasizing the role of early diagnosis and appropriate treatment in preventing long-term sequelae. We present the case of a 13-year-old girl who was admitted with left-sided central facial nerve paresis. She had a six-month history of recurrent tension headaches and unintentional weight loss. Brain MRI revealed an ischemic lesion in the right thalamus and internal capsule, with additional findings in the left thalamus and cerebellar hemispheres on follow-up imaging. The diagnostic workup revealed positive *Borrelia burgdorferi* antibodies in both the cerebrospinal fluid and serum, confirming neuroborreliosis. Causal treatment was initiated with a third-generation cephalosporin, resulting in significant clinical improvement.

Pediatric acute ischemic stroke in the course of secondary vasculitis on an infectious background appears to be the leading cause of stroke in children, which underscores the need for a thorough diagnostic evaluation targeting treatable infectious etiologies in all pediatric stroke cases. Early identification and causal treatment of such conditions, particularly neuroborreliosis, significantly improve neurological outcomes and increase the likelihood of full recovery. Therefore, potential infectious causes should not only be actively investigated but also considered when initiating empirical treatment. There is a necessity of maintaining high clinical vigilance in symptomatic patients from endemic regions presenting solely with positive Borrelia IgG serology, as such a profile does not exclude the presence of active and potentially severe neuroborreliosis.

## Introduction

Pediatric arterial ischemic stroke (PAIS) is a rare but serious condition that can result in significant neurological deficits. This report describes the case of a 13-year-old girl presenting with left-sided central facial nerve palsy and recurrent tension-type headaches, who was ultimately diagnosed with Lyme borreliosis as the underlying cause of acute ischemic stroke. Lyme borreliosis is a multisystemic zoonotic disease caused by spirochetes of the Borrelia genus, transmitted to humans via ticks of the Ixodes genus. The infection can involve the nervous system, resulting in neuroborreliosis. In the United States, Lyme disease is primarily caused by *Borrelia burgdorferi* sensu stricto, whereas in Europe, a broader spectrum of *Borrelia* genospecies, including *B. burgdorferi* sensu stricto, *B. afzelii*, and *B. garinii*, is responsible. These differences in causative agents contribute to variations in clinical manifestations and disease patterns across the two continents. Lyme arthritis is more prevalent in the United States, while Lyme neuroborreliosis, which may lead to PAIS, appears more frequently in European children, typically presenting as meningitis or isolated facial nerve palsy. Rarely, it can manifest as an acute ischemic stroke associated with secondary cerebral vasculitis [1]. By presenting this case, we aim to raise awareness of infectious etiologies, particularly neuroborreliosis pediatric stroke, emphasizing the importance of early diagnosis and targeted treatment to prevent long-term neurological sequelae.

## Case presentation

An almost 13-year-old girl was admitted to the district hospital with central paresis of the facial nerve on the left side. The child had a two-day history of facial nerve paresis. Moreover, for six months, the girl had had tension headaches occurring approximately once a week, mainly in the morning, located in the frontal area, assessed on the NRS scale as 6/10 points. Sinusitis was diagnosed, followed by the introduction of amoxicillin into the treatment, with improvement. Additionally, weakness and unintentional weight loss of approximately 7 kg were observed.

**Figure 1:**
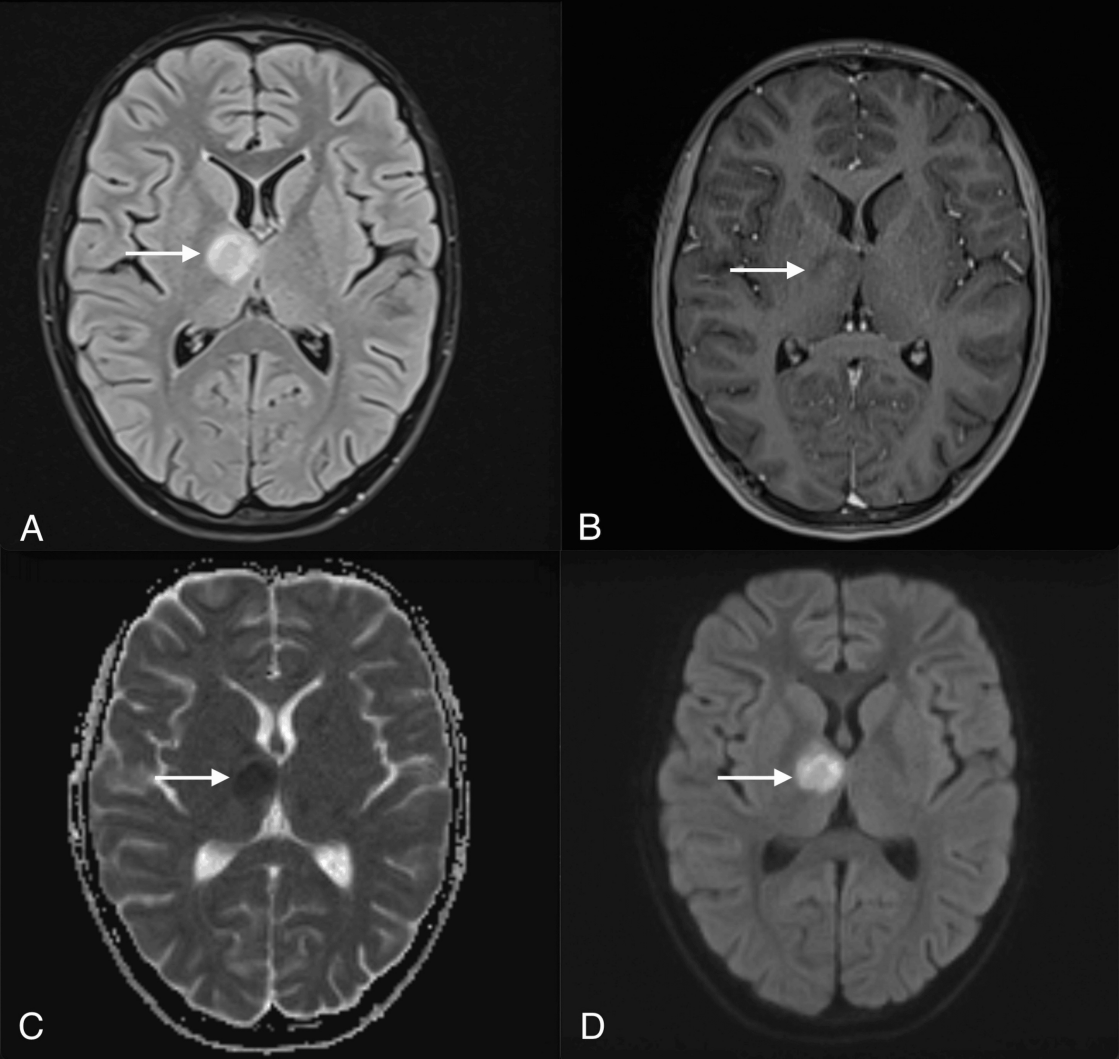
Day 5 from the onset of facial nerve palsy symptoms. Brain MRI at admission to the Pediatric Department demonstrates a pathological oval lesion in the right thalamus and internal capsule, hyperintense on T2 FLAIR (A) with trace enhancement on post-contrast T1-weighted imaging (B). Restricted diffusion is seen on the ADC map (C) and DWI (D). Arrows indicate the lesion. MRI, magnetic resonance imaging; ADC, apparent diffusion coefficient; DWI, diffusion-weighted imaging

After admission to the Pediatric Department, on the fifth day after the onset of symptoms, a magnetic resonance imaging (MRI) examination of the brain was performed. Part of the scan showed a pathological oval area measuring 17 x 15 mm with an increased T2 signal, which was limiting diffusion and had a slight mass effect and trace enhancement in the right thalamus and internal capsule (Figure 1).

Based on these observations, a stroke was suspected. The diagnostic process aimed at identifying infectious diseases was initiated. Antibodies against herpes simplex virus (HSV) were negative in the IgM class, and antibodies against Epstein-Barr virus were negative in the IgM class and positive in IgG class, with high avidity. Antibodies against *B. burgdorferi* were also determined using the ELISA method and were found to be positive in the IgG class and negative in the IgM class.

The child was transferred to the Pediatric Neurology Department. There, during the neurological examination, drooping of the left corner of the mouth at rest was observed but was hardly visible during mouth movements. Additionally, the eyelids were closed on both sides, and the forehead was wrinkled on both sides. Anisocoria (left pupil wider than right) was also observed.

In the laboratory tests, parameters of the coagulation system were determined, including the level of D-dimer and protein S. The Leiden mutation of the *F5* gene of factor V of the coagulation system was excluded. Antibodies were determined against antinuclear antibodies, anticardiolipin, lupus anticoagulant, myeloperoxidase, and proteinase 3; however, the results showed no significant deviations from the norm. Homocysteine and lipoprotein levels were normal, but the LDL cholesterol level in the lipid profile was slightly increased. The aminogram of plasma proteins was also determined.

**Figure 2:**
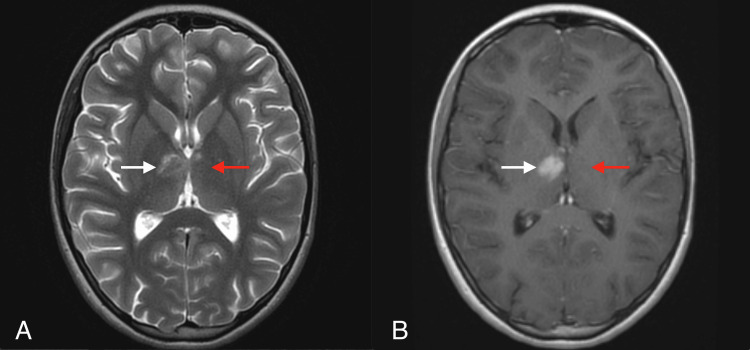
Day 12 from symptom onset. Brain MRI performed in the Pediatric Neurology Department demonstrates evolution of stroke lesions: right thalamic infarct (white arrow) and small punctate changes in the left thalamus (red arrow) on T2-weighted (A) and post-contrast T1-weighted with trace enhancement (B) images.

Chest X-ray and abdominal ultrasound imaging tests were performed, which showed no significant pathologies, and the Doppler ultrasound showed normal flows in the carotid arteries. The control MRI of the brain performed seven days after the previous examination revealed the evolution of stroke lesions: apart from the stroke focus in the right thalamus, small-point changes were described in the left thalamus and in the cerebellar hemispheres (Figure 2). Magnetic resonance angiography (MRA) revealed a developmental variant in the form of a hypoplastic left posterior communicating artery. The cerebral vessels were otherwise normal.

The child underwent a cardiological consultation, an ECG was performed (without any significant abnormalities). No structural heart defect was detected in the echocardiogram apart from abnormal structure/prolapse of the mitral valve leaflet. The diagnosis was confirmed by transesophageal ECHO and cardiac catheterization.

The treatment involved acetylsalicylic acid at a dose of 3 mg/kg/day. A reduction in the facial nerve paresis was observed. After eight days of hospitalization, the patient was discharged home. According to the parent, the girl was significantly weakened and apathetic, and a change in behavior and pathological drowsiness were observed. Lyme disease serology was determined using the Western blot method and was positive in the IgG class and negative in the IgM class.

Due to the persistence of symptoms, after receiving the results of *Borrelia* testing one month later and in the sixth week from symptom onset, the parent presented with the child to the Infectious Diseases Department. A lumbar puncture was performed, and the results of the general examination of the cerebrospinal fluid (CSF) revealed significant abnormalities (Table 1). A panel of 14 pathogens (cytomegalovirus, enterovirus, HSV-1, HSV-2, HHV-6, Paraechovirus, varicella zoster virus [VZV], E*scherichia coli*, *Haemophilus influenzae*, *Listeria monocytogenes*, *Neisseria meningitidis*, *Streptococcus agalactiae*, S*treptococcus pneumoniae*, *Cryptocccus neoformans*/*gattii*) was negative on polymerase chain reaction (PCR). CSF culture was also negative.

**Table 1:** Results of the general CSF analysis CSF, cerebrospinal fluid

Parameter	Reference values	Test	Control
Color	-	Colorless	Colorless
Clarity	-	Slightly turbid	Clear
Protein (g/L)	0.15–0.4	3.08	0.72
Glucose (mmol/L)	2.4–4.7	<0.28	1.78
Chloride (mmol/L)	113–127	116.00	121.00
Cell count (n/uL)	0–20	3,147	127

Since the results of serological tests performed before admission to the Infectious Diseases Department were inconsistent, they were repeated in a certified laboratory, with *Borrelia* IgM antibodies being positive in both serum and CSF at low titters: IgM serum 20.74 BBU/mL (negative ≤9.00; positive ≥11.00) and IgM CSF 13.16 BBU/mL (negative ≤5.00; positive >5.00). Borrelia IgG antibodies were critically positive at a titer of 65,000 BBU/mL in CSF (positive result >5.00) and in serum at 7,632 BBU/mL (positive result >11.00), and index of intrathecal synthesis of *Borrelia* spp. antibodies was 6.96 (positive >1.3).

Due to the diagnosis of neuroborreliosis, ceftriaxone was administered at a maximum dose of 2 grams twice daily (100 mg/kg). Treatment started 42 days after the onset of symptoms such as facial nerve palsy.

The diagnostic evaluation was complemented by testing for other infectious diseases that may play a significant role in the etiology of stroke. Serological testing revealed significantly elevated SARS-CoV-2 IgG antibodies, with negative IgM antibodies (the patient had not been vaccinated). HIV COMBO test was non-reactive. Mycoplasma testing was negative for both IgM and IgG. Antibodies against tick-borne encephalitis (TBE) were not determined (the patient was vaccinated).

Due to the atypical results of the CSF general test, a follow-up lumbar puncture was performed on day 26 of treatment. In the control CSF examination, there was a significant decrease in cytosis to 127/uL, in a protein level to 0.72 g/L, and glucose to 1.78 mmol/L (Table 1). Due to the lack of normalization of these parameters, the decision was made to extend the antibiotic therapy. The treatment ended after 32 days.

**Figure 3:**
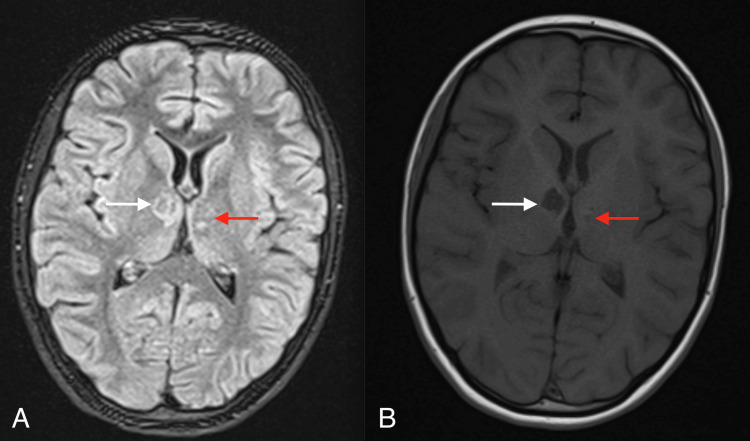
Week 18 from symptom onset. Follow-up brain MRI performed in the Infectious Diseases Department shows an area of malacia in the basal ganglia and thalamus (white arrow) and small foci of porencephaly in the left basal ganglia (red arrow) on T2 FLAIR (A) and post-contrast T1-weighted imaging (B).

After initiating ceftriaxone treatment, a significant improvement in the patient's condition and well-being was observed. The weakness and apathy disappeared, the girl became active and cheerful, and her appetite returned. She gained 3.3 kg during hospitalization. When discharged from the hospital, the girl did not show any abnormalities in the neurological examination: meningeal and focal symptoms were absent. The patient also did not complain about mood disorders, headaches, or lack of appetite. The follow-up MRI of the head revealed an area of malacia in the basal ganglia and thalamus, as well as single small foci of porencephaly in the left basal ganglia (Figure 3).

## Discussion

Stroke, as defined by the World Health Organization (WHO), is "a clinical syndrome characterized by the sudden onset of symptoms of focal or generalized disturbances of cerebral function which persist, if not fatal, for longer than 24 hours and have no other cause than vascular" [2].

In the pediatric population, compared to adults, it is a relatively rare disease, with an estimated frequency of 3-13 cases per 100,000 per year. Strokes in newborns constitute a significant percentage, with an overall incidence of up to one in 1,000 live births. [3,4]. These data may be an underestimation due to the insufficient diagnosis of this disease.

Despite the low incidence of stroke, it is a significant cause of death in the pediatric population (cerebrovascular diseases are among the 10 most common causes of death), and in the absence of death, they often result in serious complications and neurological deficits such as movement disorders, speech disorders, intellectual functions, epileptic seizures, and psycho-motor development disorders in newborns and infants [5].

In the adult population, modifiable risk factors for stroke include hypertension, diabetes, cardiac diseases (especially atrial fibrillation), stimulants (alcohol, cigarettes), and non-modifiable factors: gender, age, and genetic factors.

In the pediatric population, risk factors vary and are listed in Table 2 [6-9].

**Table 2:** Risk factors for PAIS CADASIL, cerebral autosomal dominant arteriopathy with subcortical infarcts and leukoencephalopathy; CARASIL, cerebral autosomal recessive arteriopathy with subcortical infarcts and leukoencephalopathy; CARASAL, cathepsin a-related arteriopathy with strokes and leukoencephalopathy; MELAS, mitochondrial encephalomyopathy, lactic acidosis, and stroke-like episodes; HIV, human immunodeficiency virus; EBV, Epstein–Barr virus; CMV, cytomegalovirus

Risk factors	
Heart disease	Ventricular septal defect, atrial septal defect, patent ductus arteriosus, complex congenital heart defects, valvular heart disease, rheumatologic diseases, endocarditis, myocarditis, cardiac arrhythmias
Systemic vascular diseases	Hypertension, diabetes, hypernatremia, hyperlipoproteinemia
Secondary cerebral vasculitis	Systemic connective tissue diseases and systemic autoinflammatory diseases: Systemic lupus erythematosus, Kawasaki disease, Takayasu arteritis, Polyarteritis nodosa
Vasculopathies	Moyamoya disease (primary and secondary), Ehlers-Danlos syndrome
Genetic vasculopathies	CADASIL, CARASIL, CARASAL
Diseases associated with vasoconstriction	Migraine, psychoactive substances (cocaine)
Coagulopathies and hematological diseases	Deficiencies of protein C and S, antithrombin III, antiphospholipid antibodies, thrombocytopenic purpura, thrombocytosis, polycythemia, hemoglobinopathies, leukemias
Inborn errors of metabolism	MELAS syndrome, homocystinuria, Fabry disease, lipid metabolism disorders with hyperlipidemia
Injuries	Intracranial injuries, carotid artery injuries
Infectious diseases	Chickenpox, HIV, meningitis, Lyme disease, COVID-19, influenza, Mycoplasma pneumoniae, Treponema pallidum, Mycobacterium tuberculosis, EBV, CMV

In many patients, despite extensive diagnostics, the risk factor cannot be clearly determined, and only a probable cause can be indicated. Often, several factors can be identified in one patient [10,11].

Stroke symptoms depend on the location of the stroke focus and its extent, especially in older children. They usually include paralysis and hemiparesis, cranial nerve palsies, aphasia, convulsions, headaches, and impaired consciousness. In newborns, the symptoms are usually less specific and include circulatory and respiratory disorders, disturbances of consciousness, and convulsions. In the presented patient, MRI showed lesions in the right thalamus and internal capsule. Clinical symptoms of thalamic damage depend on which part of it has been damaged and include memory, behavior, and personality disorders, learning disorders, visual disturbances, and speech disorders, as well as Dejerine-Roussy syndrome. Infarctions of different portions of the internal capsule also have varied clinical presentations. They may manifest as hemiparesis and hemisensory deficits collateral to the location of the lesion, weakness of facial and tongue muscles, dysarthria, visual disturbances, and hearing deficits, as well as confusion, concentration disorders, and agitation. Symptoms of facial nerve paralysis observed in patients are most consistent with lacunar infarction in the genu of the internal capsule. Anisocoria also may occur in internal capsule stroke, but it is not a symptom unique to this type of stroke. Behavioral changes reported by the parent at admission may be attributable to thalamic damage. Dominant symptoms, such as fatigue, mood disorders, headaches, and weight loss, which had persisted for several weeks, were most likely related to ongoing small vessel inflammation, leading to secondary vasculitis, which is the leading cause of stroke in the course of neuroborreliosis as well as other infectious diseases such as Mycoplasma pneumoniae or post-varicella angiopathy. Additionally, it is characteristic that most strokes associated with neuroborreliosis are located in areas of the brain supplied by the posterior cerebral circulation. In the case described, the main stroke focus was the thalamus supplied by the posterior cerebral artery and the posterior communicating artery.

Neuroimaging tests are necessary to diagnose a stroke; due to their widespread availability, in patients with symptoms of ischemic stroke, computer tomography (CT) is usually performed first, but it may not show ischemic changes, especially in the first hours. A much more valuable test is a brain MRI, especially with the diffusion option. Black-blood MRI sequences and MRA are performed to identify vascular changes.

It is also important to consider infectious agents in the examination of CSF. Polymorphonuclear pleocytosis is not characteristic of neuroborreliosis. Therefore, PCR and CSF culture were performed, which were negative. The patient had low serum inflammatory markers and a normal blood count and was in good general condition and afebrile. Additionally, with high titers of antibodies against Lyme disease, there is no basis to suspect bacterial meningitis of an etiology other than *Borrelia*. There are reports in the literature of strokes associated with VZV infection occurring up to 12 months after onset [12-14]. However, in our patient, there was no history of symptomatic VZV infection, and serological assays and PCR testing of the CSF were negative. Serological testing revealed markedly elevated anti-SARS antibody titers in the patient; however, in the pediatric population, unlike in adults, the association between prior COVID-19 and the occurrence of stroke is not well established [15,16]. Cases of stroke in the course of neuroborreliosis have also been described [17-19]. It occurs more frequently in Europe, where *Borrelia *genospecies, including *B. burgdorferi *sensu stricto, *B. afzelii*, and *B. garinii*, are the primary causative agents of Lyme neuroborreliosis, which may subsequently lead to PAIS. [1].

According to the 2023 Polish Society of Epidemiologists and Infectious Diseases Physicians (PTEILCHZ) guidelines, treatment for neuroborreliosis should last two to three weeks [20]. There are also guidelines requiring antibiotic therapy for some forms of neuroborreliosis for up to four weeks, such as those from the Spanish Society of Infectious Diseases and Clinical Microbiology (SEIMC), Spanish Society of Neurology (SEN), Spanish Society of Immunology (SEI), Spanish Society of Pediatric Infectiology (SEIP), Spanish Society of Rheumatology (SER), and Spanish Academy of Dermatology and Venereology (AEDV) [21]. According to the guidelines, there are no indications for a follow-up lumbar puncture. In our case, a follow-up lumbar puncture was undertaken because of several atypical features, including neutrophilic pleocytosis, markedly elevated anti-*Borrelia* antibody titers in the CSF, the relatively delayed initiation of treatment, and a clinical course complicated by stroke. Although the patient demonstrated a striking clinical improvement, normalization of CSF parameters had not yet been achieved. Given these considerations, the decision was made to extend antimicrobial therapy beyond the standard duration.

In the case of the patient hospitalized in the Pediatric Department, extensive diagnostics did not find any factors contributing to the occurrence of PAIS. Presentation of IgM antibodies against *B. burgdorferi* in both serum and CSF and very high titers of IgG antibodies (in the CSF 13,000 times above the norm), strongly positive index of intrathecal synthesis, and an obvious improvement in the child's condition after the introduction of third-generation cephalosporins to the treatment, considering that corticosteroids were not employed at any stage of the patient’s treatment, seem to indicate that neuroborreliosis was the most important factor in the occurrence of ischemic stroke associated with secondary cerebral vasculitis in this child.

## Conclusions

PAIS in the course of secondary vasculitis on an infectious background appear to be the leading cause of stroke in children, which underscores the need for a thorough diagnostic evaluation targeting treatable infectious etiologies in all pediatric stroke cases. Early identification and causal treatment of such conditions, particularly neuroborreliosis, significantly improve neurological outcomes and increase the likelihood of full recovery. Therefore, potential infectious causes should not only be actively investigated but also considered when initiating empirical treatment. There is the necessity of maintaining high clinical vigilance in symptomatic patients from endemic regions presenting solely with positive Borrelia IgG serology, as such a profile does not exclude the presence of active and potentially severe neuroborreliosis.
